# Biomechanical evaluation of cervical disc replacement with a novel prosthesis based on the physiological curvature of endplate

**DOI:** 10.1186/s13018-018-0748-7

**Published:** 2018-02-27

**Authors:** Jigang Lou, Yuanchao Li, Beiyu Wang, Yang Meng, Quan Gong, Hao Liu

**Affiliations:** 1 Department of Orthopedics, West China Hospital, Sichuan University, 37 Guoxue Road, Chengdu, Sichuan 610041 China; 2 Department of Biomechanical Research Laboratory, Shanghai Jiao Tong University, Shanghai, China

**Keywords:** Biomechanics, Cervical disc replacement, Intradiscal pressure, Disc prosthesis, Range of motion

## Abstract

**Background:**

Most of the current available cervical disc prostheses present a flat surface instead of an arcuate surface which is most similar to the morphology of cervical endplate. Therefore, we designed a novel prosthesis (Pretic-I, Trauson) based on the physiological curvature of the cervical endplate. Biomechanical evaluation of cervical disc replacement (CDR) with this novel prosthesis was performed and compared with the Prestige LP prosthesis.

**Methods:**

Three motion segments of 18 cadaveric cervical specimens (C2-C7) were evaluated with a 75 N follower load. Overall, the biomechanics of three models, intact specimen, CDR with the novel prosthesis and CDR with the Prestige LP prosthesis, were studied to gain insight into the effective function of the novel prosthesis. The range of motion (ROM) of all three segments and intradiscal pressure (IDP) on adjacent levels were measured and analysed.

**Results:**

Compared to the intact condition, the ROM of all three segments showed no significant difference in the replacement group. Moreover, there was also no significant difference in the ROM between the two prostheses. Besides, the IDP on the cranial adjacent level showed no obvious difference between the two prostheses; nevertheless, the IDP on the caudal adjacent level of the novel prosthesis was significantly less than the Prestige LP prosthesis.

**Conclusions:**

In summary, the novel disc prosthesis was effective to maintain the ROM at the target segment and adjacent segments. Besides, CDR with the novel prosthesis could reduce the IDP on the caudal adjacent level to a certain extent, compared with the Prestige LP prosthesis.

**Electronic supplementary material:**

The online version of this article (10.1186/s13018-018-0748-7) contains supplementary material, which is available to authorized users.

## Background

Cervical disc replacement (CDR) is a relatively new technology in spinal surgery, which allows the preservation of the mobility at the implanted segment, and could reduce the stress sustained by adjacent levels and slow down the progression of degeneration of adjacent segments, compared with fusion [1–3]. However, it is still unknown that whether CDR reduces rates of adjacent segment degeneration, compared with the natural history of the disease [4, 5]. Moreover, artificial disc prostheses should be constantly improved and designed more scientifically, in order to reduce the prosthesis-related complications including subsidence, loosening, migration, dislocation, device wear and heterotopic ossification which have been widely reported [6, 7].

To our knowledge, most of the current available cervical disc prostheses with various design concepts present a flat surface instead of an arcuate surface. However, as the morphology of inferior endplates of the cervical spine is mainly concave [8, 9], the mismatch between the prosthesis surface and the endplate geometry gives rise to an inadequate load distribution across the prosthesis-endplate interface, which may be responsible for prosthesis subsidence [10]. Besides, size mismatch in the current available cervical disc prostheses is another noteworthy issue, since an undersized prosthesis is unable to cover the peripheral marginal zones of the endplate whose biomechanical strength is much larger than that of the central areas so as to increase the probability of prosthesis subsidence [11–13]. Therefore, according to the anatomy of cervical vertebra and the people’s physical size of cervical disc in China [14, 15], we designed a novel artificial disc prosthesis (Pretic-I, Trauson) based on the physiological curvature of cervical endplate, with an advantage of increasing the contact area between the prosthesis and endplate to disperse the axial load.

In this study, a novel cervical disc prosthesis is tested in vitro, whose biomechanical behaviour is compared with intact cervical spines, and implanted spines with the Prestige LP (Medtronic, Memphis, TN, USA) prosthesis whose surface is flat. Our aim is to test whether or not CDR with the novel prosthesis preserves, or is most similar to, the normal range of motion (ROM) of the cervical spine, and sustains the intradiscal pressure (IDP) on adjacent segments. A secondary objective is to analyse the biomechanical differences between the two prostheses.

## Methods

### Device design

**Fig. 1:**
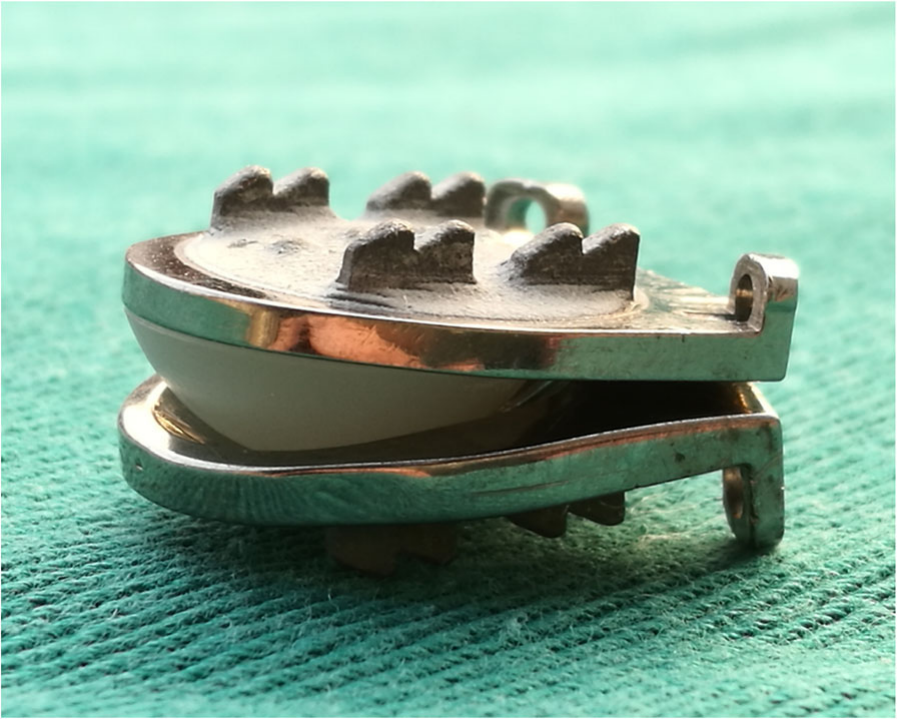
Pretic-I disc prosthesis. It is composed of a superior plate, an inferior plate and an inlay

The Pretic-I cervical disc prosthesis consists of a ball-in-socket design with superior and inferior plates (Ti6A14V) and a hemispherical core made of ultra-high-molecular-weight polyethylene (UHMWPE) (Fig. 1), making its biocompatibility and wear resistance much better. The spherical superior articular surface and the oval inferior articular surface can allow the superior plate to move back and forth along a slot in the horizontal direction. Hence, the centre of rotation of the prosthesis is not fixed. The back surface of each plate has two rows of dentate crests to improve the initial stability of the prosthesis and avoid implant migration, and it is sprayed with a hydroxyapatite coating to allow bone in-growth to the implant. The dimensions of the Pretic-I prosthesis have been reported in a previous study [16].

### Specimen preparation

Eighteen fresh-frozen human cadaveric cervical spines (C2-C7) that came from donors were used in this biomechanical test. The specimens were collected from 11 males and 7 females aged between 28 and 72 years; their height ranged from 155 to 178 cm, and their mass ranged from 49 to 78 kg. Radiographs were taken to ensure that no specimen had obvious flaws, such as fractures, deformities, tumours, metastatic disease, osteoporosis or disc degeneration (osteophytes, disc space narrowing or facet hypertrophy). Each specimen was then kept frozen for less than 6 months in double-sealed bags at − 20 °C. All procedures for specimen preparation were similar to a previous study [17]. In preparation for biomechanical testing, all specimens were thawed to room temperature. Care was taken to preserve all ligamentous attachments, only the muscular and fatty tissues were removed. All specimens were moistened with 0.9% NaCl solution to prevent desiccation during testing. The study was approved by the ethics committee of the West China Hospital, Sichuan University. Specimens were prepared for testing in a polysegmental set-up. For the stabilisation, the proximal (C2) and distal (C7) ends of the specimen were embedded in polymethylmethacrylate in cylindrical aluminium fixtures. C7 was prepared for additional stabilisation by partially inserting three perpendicular screws into the exposed end plates.

### Biomechanical testing apparatus

**Fig. 2:**
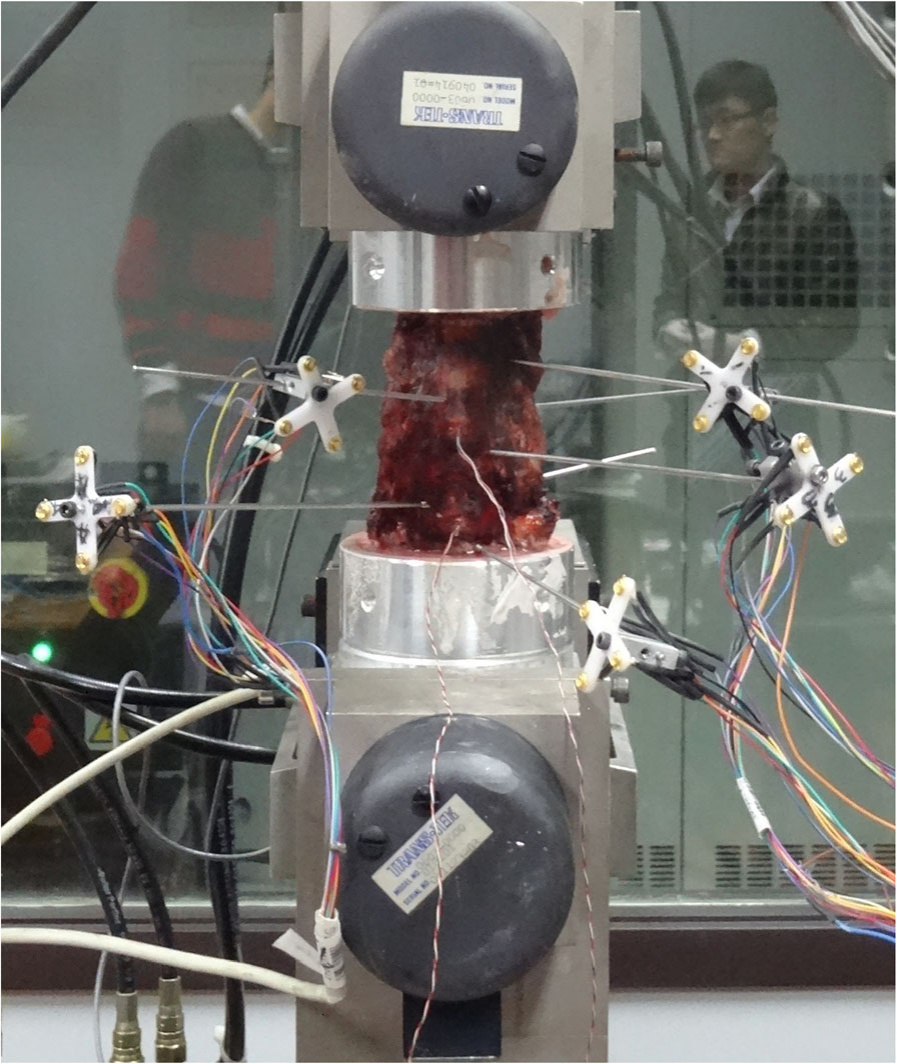
Anterior view of an intact specimen. Each Kirschner pin connected four optical markers to each vertebral body of the specimen. The pressure measuring sensors (Model 060) were inserted into the nucleus at the centre of the disc in C4/5 and C6/7 segment

Biomechanical testing was performed using multi-degree of freedom servo-hydraulic testing system (MTS Bionix 858, MTS Corporation, Minneapolis, MN), which was capable of applying pure moments about three axes to simulate flexion-extension, lateral bending and axial rotation under load control, as described previously [17, 18]. A 2.0-Nm maximum moment loading at a rate of 0.2 Nm s^−1^ was applied to the proximal end (C2) of the specimen, whereas the distal portion (C7) remained fixed to the socket of the apparatus. During each mode of loading, a constant compressive follower load of 75 N was applied, which approached the physiological conditions prevailing in the cervical spine [19]. For evaluating the total ROM and segmental ROM of the specimens, an optical tracking system (Polaris Northern Digital Incorporation, Ontario, Canada) was used. Moreover, a Kirschner pin connected four optical markers to each vertebral body of the specimen. The pressure measuring sensors Model 060 (Precision Measurement Company, Ann Arbor, Michigan, USA) were inserted into the nucleus at the centre of the disc in the C4/5 and C6/7 segments under radiograph control to measure the IDP (Fig. 2) [20]. While each load was applied, voltage outputs from the pressure sensors were recorded continuously. In addition, each test was repeated for three loading cycles. Moreover, the data from the third cycle was used for analysis.

### Surgical procedure

**Fig. 3:**
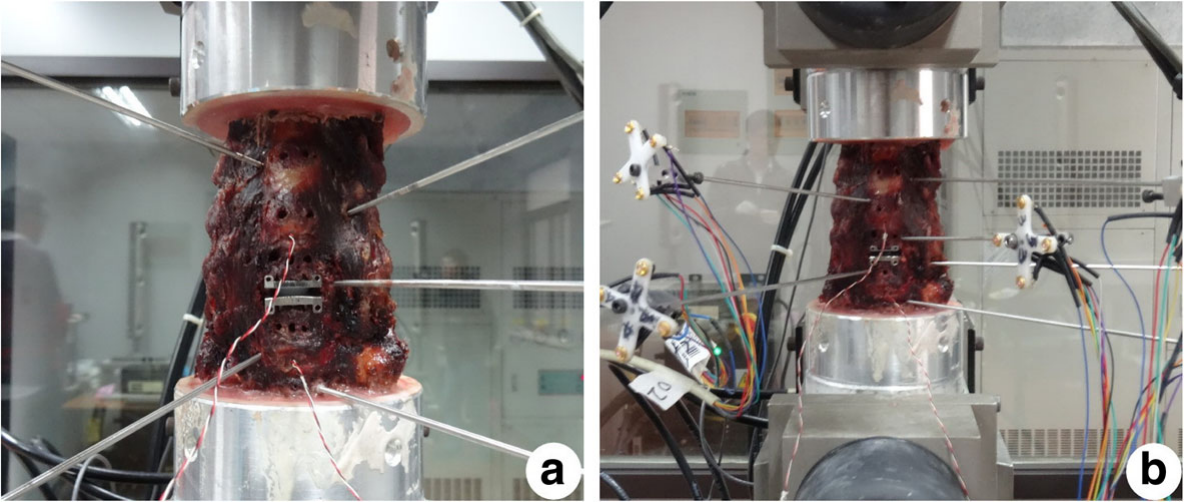
Implanted specimens. **a** CDR with the novel prosthesis Pretic-I. **b** CDR with the Prestige LP prosthesis

**Fig. 4:**
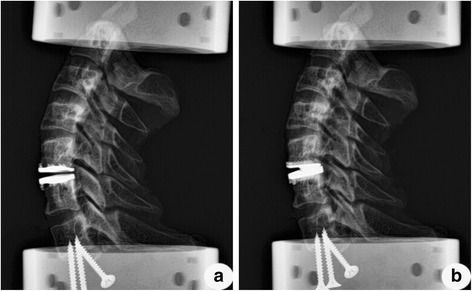
Radiographs of two different implanted specimens. The correct position of the novel prosthesis Pretic-I (**a**) and the Prestige LP prosthesis (**b**)

Eighteen specimens were divided into three groups (groups 1, 2 and 3), with six specimens per group. A complete disc discectomy of C5/6 was performed in all cervical spines of groups 2 and 3. The posterior longitudinal ligament was routinely resected. The end plates were flattened to an appropriate extent using a curette and a high-speed burr. CDR was performed according to the manufacturer’s recommended tools and procedures. Using a guide tool, reference pins were inserted into the vertebral bodies above and below the target segment, leaving holes in the vertebral bodies. Trial sizes were used to assess the appropriate size of the prosthesis. The optimal prosthesis was then attached as a single unit to an insertion tool and driven into place with a hammer. In group 2, the Pretic-I prosthesis was inserted at the C5/6 segment (Fig. 3a), while the Prestige LP prosthesis was implanted at the same level in group 3 (Fig. 3b). All specimens were measured and analysed. Radiographs were taken to check the correct position of all implanted disc prostheses (Fig. 4).

### Statistical analysis

Mean values and standard deviations were determined for each parameter. SPSS software (Version 19.0, SPSS Inc., Chicago, IL, USA) was used for statistical analysis. All data were analysed using one-way analysis of variance followed by Holm-Sidak tests to determine whether or not the outcome measures were significantly different among the intact condition, CDR with the Pretic-I prosthesis and CDR with the Prestige LP prosthesis. A value of *P* < 0.05 was considered statistically significant.

## Results

### Total ROM and segmental ROM

**Fig. 5:**
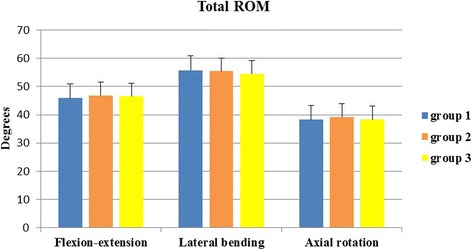
Total ROM. Total ROM of intact (group 1), CDR with the novel prosthesis Pretic-I (group 2) and CDR with the Prestige LP prosthesis (group 3) in all three motion directions

The differences in total ROM among the three groups were not statistically significant in all three motion directions with the following values: for flexion-extension 45.93° ± 3.43° in group 1, 46.86° ± 2.76° in group 2 and 46.49° ± 2.88° in group 3; for lateral bending 55.80° ± 3.91° in group 1, 55.44° ± 3.91° in group 2 and 54.52° ± 5.10° in group 3; for axial rotation 38.24° ± 4.96° in group 1, 39.25° ± 3.72° in group 2 and 38.33° ± 4.53° in group 3. The mean total ROM in flexion-extension, lateral bending and axial rotation was always recorded at the maximum loading of plus or minus 2 Nm. The total ROM of the three groups underwent minor changes in all three directions of motion (*P* > 0.05) (Fig. 5).

**Fig. 6:**
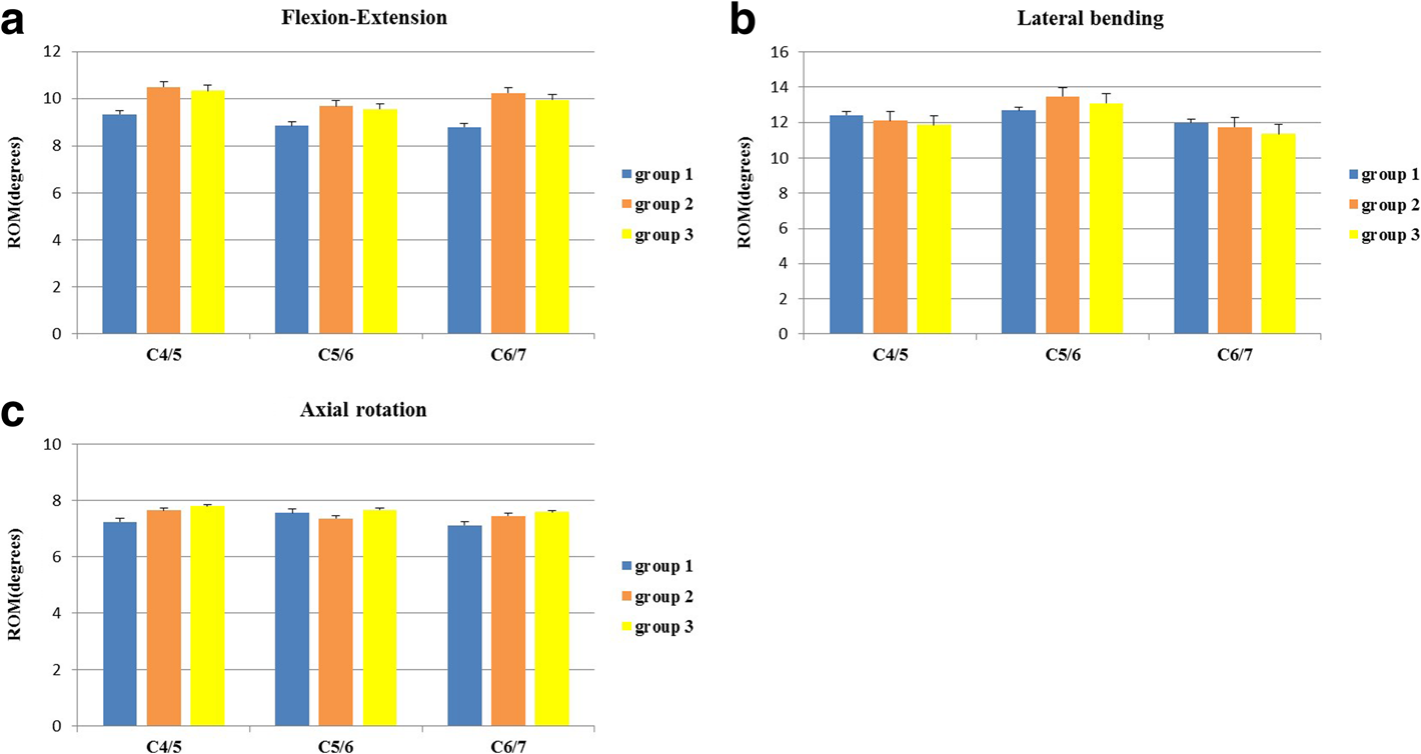
Segmental ROM. Segmental ROM of the three groups in flexion-extension (**a**), lateral bending (**b**) and axial rotation (**c**)

The mean values of segmental ROM at C4/5, C5/6 and C6/7 in all three directions of motion in each of the three groups are shown in Table 1. In a manner similar to the total ROM, the differences in segmental ROM among the three groups were also not statistically significant in all three directions of motion (*P* > 0.05) (Fig. 6).

**Table 1:** The ROM at the implanted segment and adjacent segments in flexion-extension, lateral bending and axial rotation

Segment	Flexion-extension (°)			*F*	*P*	Lateral bending (°)			*F*	*P*	Axial rotation (°)			*F*	*P*
	Group 1	Group 2	Group 3			group1	group2	group3			Group 1	Group 2	Group 3		
C4/5	9.32±1.41	10.49 ± 1.57	10.33 ± 1.88	0.92	0.42	12.41 ± 1.58	12.10 ± 1.43	11.85 ± 1.50	0.21	0.81	7.23±1.47	7.65±1.26	7.79±1.19	0.30	0.75
C5/6	8.83±1.51	9.68±1.73	9.55±1.73	0.46	0.64	12.67 ± 1.60	13.46 ± 2.11	13.09 ± 1.98	0.25	0.78	7.56±1.31	7.36±1.20	7.66±1.57	0.08	0.93
C6/7	8.78±1.41	10.24 ± 1.51	9.94±1.74	1.47	0.26	11.96 ± 1.53	11.74 ± 1.35	11.36 ± 1.43	0.26	0.77	7.12±1.55	7.45±1.41	7.59±1.20	0.18	0.84

The ROM was expressed as mean ± standard deviation. Group 1, intact spine; group 2, CDR with the novel prosthesis; group 3, CDR with the Prestige LP prosthesis

### Pressure analysis

**Fig. 7:**
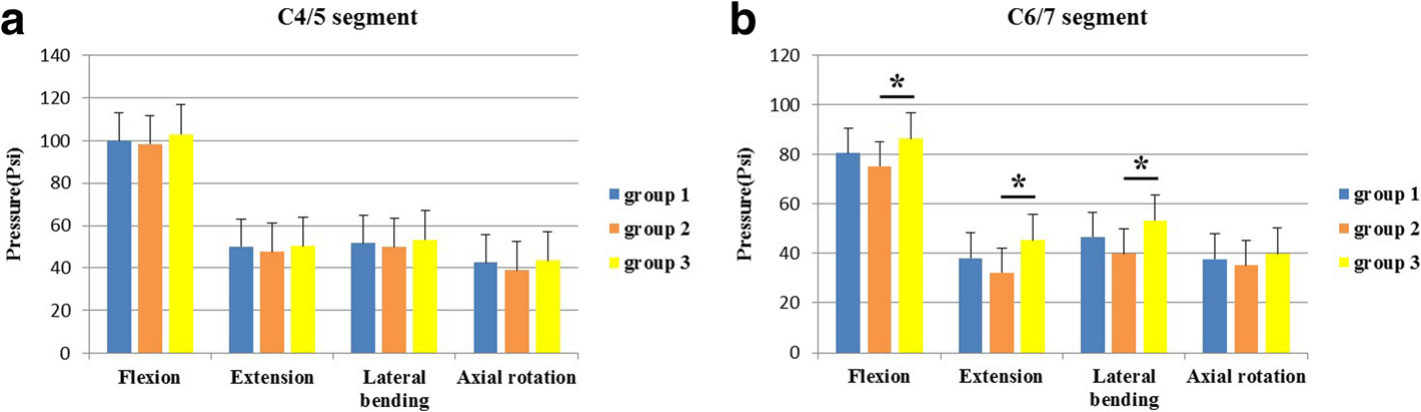
IDP on adjacent segments. **a** IDP on segment C4/5 of the three groups exhibited no significant differences in all three motion directions. **b** IDP on segment C6/7 of the novel prosthesis (group 2) was significantly lower than those on the Prestige LP prosthesis (group 3) in flexion, extension and lateral bending, but not during axial rotation. Statistically significant differences are denoted by * (*P* < 0.05), with bars connecting the corresponding columns

The differences in the IDP on segment C4/5 among the three groups were not statistically significant in all three directions of motion (*P* > 0.05) (Fig. 7a). In addition, the differences in the IDP on segment C6/7 between group 1 and the replacement group (groups 2 or 3) were also not statistically significant in all three directions of motion. However, when comparing groups 2 and 3 directly, we found significant differences in the IDP on segment C6/7 in flexion, extension and lateral bending but not under axial rotation (Fig. 7b). The mean IDP in group 2 was significantly lower than those in group 3 in flexion, extension and lateral bending (*P* = 0.038 in flexion, *P* = 0.039 in extension and *P* = 0.043 in lateral bending). The mean IDP in group 2 was also lower than those in group 3 during axial rotation, but the differences were not statistically significant (*P* > 0.05) (Additional file 1).

## Discussion

CDR is a successful, promising, non-fusion technique aimed at restoring the disc space height and spine kinematics. Previous studies [21–23] have demonstrated that CDR confers more benefits than anterior cervical discectomy and fusion, the gold standard technique. Prosthetic devices of the correct sizes and supported by scientific design processes are crucial to the success and long-term survival of CDR. Therefore, this novel artificial disc prosthesis warrants further investigation, as it is designed based on the physiological curvature of the cervical endplate and the people’s anatomy of the cervical vertebra in China. Human spinal specimens are often used for in vitro testing. After being implanted into the segments, we also tested the ROM of all three segments and IDP on adjacent segments to assess the function of this novel prosthesis, and in comparison with the Prestige LP prosthesis.

For the biomechanical testing, although the application of a cervical follower load would decrease the ROM under lateral bending of functional spinal units [24], we still adopted a 75 N follower load to increase clinical practicability. Furthermore, there has always been a dispute about using a load-controlled, or displacement-controlled, protocol during biomechanical testing [25]. We deemed it to be the case that the load-controlled testing mode may better reflect the axial load from the weight of the head and muscle forces in the neck, making the testing of spinal specimens a better representation of prevailing physiological functional conditions. Consequently, we adopted the load-controlled testing mode for these biomechanical tests.

In the present study, we tested the ROM at the target segment and adjacent segments under three conditions (intact spine and CDR with two types of artificial disc prostheses). In addition, the IDP on adjacent segments was also analysed. There were minor changes in ROM after CDR with the novel prosthesis and the Prestige LP prosthesis (*P* > 0.05). Similar to previous studies, the differences in ROM at target segment between the intact spine and CDR with the novel prosthesis were not statistically significant; the ROM at adjacent segments after CDR with the novel prosthesis also approached the values of the intact spine without significant differences between them [26, 27]. Therefore, the novel artificial disc prosthesis was similar to the Prestige LP disc prosthesis in mimicking the motion function of a normal cervical disc. With regard to IDP, the superior IDP after CDR with two types of artificial disc prostheses exhibited no significant difference; however, the inferior IDP after CDR with the novel prosthesis was significantly lower than with the Prestige LP disc prosthesis in almost all situations, except axial rotation. During axial rotation, the inferior mean IDP after CDR with the novel prosthesis was slightly lower than with the Prestige LP disc prosthesis, which may be statistically significant given a larger sample size. The result of the pressure analysis demonstrated that the novel artificial disc prosthesis could reduce the IDP on the inferior segment to some extent, compared with the Prestige LP prosthesis.

The above results may be mainly attributed to the differences between the two disc prostheses. First, compared with the Prestige LP prosthesis with a flat surface, the novel prosthesis is designed based on the physiological curvature of the cervical endplate. The arcuate surface of the novel prosthesis can provide a greater effective contact area between the prosthesis and cervical endplate, in order to disperse the axial load more evenly. Second, the novel prosthesis is designed according to the anatomy of cervical vertebra and the people’s physical size of the cervical disc in China [14, 15], providing a better size match between the prosthesis and cervical vertebra. Size match between the prosthesis and cervical vertebra can not only provide a greater contact area between the prosthesis and cervical endplate but also can cover the peripheral marginal zones of cervical endplate which provide a much stronger support than the central areas [11–13]. However, one previous study [28] investigated the most common available artificial disc prostheses and found that 53.5% of the largest device footprints were smaller in their anterior-posterior diameter, and 51.1% in the mediolateral diameter were smaller than the cervical endplate diameters. Again, compared with the Prestige LP prosthesis with a metal-on-metal design, the novel prosthesis with a metal-on-polymer design possesses much better wear resistance and stress cushioning effect [16, 29]. Thus, given the same loading from the superior vertebral body, the novel prosthesis can disperse the axial load more evenly, resulting in a smaller pressure on the inferior segment, compared with the Prestige LP prosthesis.

With regard to the IDP measurement in the fresh-frozen cadaveric specimens, there are several aspects that should be expounded. First, there is no doubt that the fresh cadaveric specimens are the optimal choice for in vitro biomechanical testing. Second, freezing may affect the IDP measurement, especially multiple freeze/thaw cycles, as IDP is based on the hydrostatic behaviour of the nucleus pulposus [30]. Again, repeated measurements (up to ten) on a single specimen at different testing conditions do not significantly affect the IDP [30]. However, factors affecting IDP measurement during in vitro biomechanical testing are complex and diverse; further investigations are still needed.

As for the deficiency of any in vitro biomechanical test using a cadaveric cervical spine, our study was mainly devoted to the investigation of the extent of motion without considering the quality, therefore, and could not reflect the long-term effect of CDR. Besides, as the human cadaver specimens were difficult to obtain, the sample size was small. Therefore, further studies with a larger sample size are still needed to evaluate more comprehensively the function of this novel prosthesis.

## Conclusions

In conclusion, CDR with the novel disc prosthesis was effective to maintain the ROM at the target segment and adjacent segments. Besides, CDR with the novel prosthesis could reduce the IDP on the caudal adjacent level to a certain extent, compared with the Prestige LP prosthesis.

## Additional file

Additional file 1: The original data on the ROM and IDP of the cadaveric cervical specimens. (XLSX 16 kb)

## Abbreviations

CDR: Cervical disc replacement
ROM: Range of motion
UHMWPE: Ultra-high-molecular-weight polyethylene
